# Infections after fiducial marker implantation for prostate radiotherapy: are we underestimating the risks?

**DOI:** 10.1186/s13014-015-0347-2

**Published:** 2015-02-13

**Authors:** Jasmin Loh, Katie Baker, Swetha Sridharan, Peter Greer, Chris Wratten, Anne Capp, Sarah Gallagher, Jarad Martin

**Affiliations:** Department of Radiation Oncology, Calvary Mater Newcastle Hospital, Edith St, Waratah, NSW 2298 Australia; Faculty of Health and Medicine, University of Newcastle, Callaghan, NSW Australia; Medical Physics Research Group, School of Mathematical and Physical Sciences, Faculty of Science & IT, The University of Newcastle & Centre for Clinical Radiation Research, Calvary Mater Newcastle, Waratah, NSW Australia

**Keywords:** Gold fiducial markers, Image guided radiotherapy, Infective complications, Prostate radiotherapy, Transrectal ultrasound guided

## Abstract

**Background:**

The use of gold fiducial markers (FM) for prostate image-guided radiotherapy (IGRT) is standard practice. Published literature suggests low rates of serious infection following this procedure of 0-1.3%, but this may be an underestimate. We aim to report on the infection incidence and severity associated with the use of transrectally implanted intraprostatic gold FM.

**Methods:**

Three hundred and fifty-nine patients who underwent transrectal FM insertion between January 2012 and December 2013 were assessed retrospectively via a self-reported questionnaire. All had standard oral fluoroquinolone antibiotic prophylaxis. The patients were asked about infective symptoms and the treatment received including antibiotics and/or related hospital admissions. Potential infective events were confirmed through medical records.

**Results:**

285 patients (79.4%) completed the questionnaire. 77 (27.0%) patients experienced increased urinary frequency and dysuria, and 33 patients (11.6%) reported episodes of chills and fevers after the procedure. 22 patients (7.7%) reported receiving antibiotics for urinary infection and eight patients (2.8%) reported hospital admission for urosepsis related to the procedure.

**Conclusion:**

The overall rate of symptomatic infection with FM implantation in this study is 7.7%, with one third requiring hospital admission. This exceeds the reported rates in other FM implantation series, but is in keeping with the larger prostate biopsy literature. Given the higher than expected complication rate, a risk-adaptive approach may be helpful. Where higher accuracy is important such as stereotactic prostate radiotherapy, the benefits of FM may still outweigh the risks. For others, a non-invasive approach for prostate IGRT such as cone-beam CT could be considered.

**Electronic supplementary material:**

The online version of this article (doi:10.1186/s13014-015-0347-2) contains supplementary material, which is available to authorized users.

## Background

Daily image guidance is a crucial component in the delivery of dose escalated external beam radiotherapy for prostate cancer. Image guided radiotherapy (IGRT) using gold fiducial markers is standard practice in many centres. Despite their adoption into routine practice, there is a lack of prospective evidence on the safety profile of the implantation of the fiducial markers. Most of the studies examining the complications of fiducial markers are done through a questionnaire, with the information collected retrospectively. These studies have reported low rates of infective complications with this procedure [1-4]. However there is a high degree of bias associated with survey studies. The results are dependent on a multitude of factors including the participant response rates, recall bias, the questions being asked and the time frame in which they are presented to the patient.

There is a large and often prospectively collated literature relating to the septic complications associated with transrectal prostate biopsies and the potential risk factors [5-10]. Given the similarity between this procedure and the fiducial marker implantation with both being performed under transrectal ultrasound (TRUS) guidance, it is likely that we can draw some inferences from the results of the more mature prostate biopsies dataset. These suggest a rate of urosepsis of approximately 3% and an increasing incidence of multidrug resistant infections [7]. This has been used to promote alternative approaches such as transperineal biopsy.

Apart from the potential complications related to the procedure, the fiducial markers do incur some financial cost. With non-invasive methods such as cone beam CT (CBCT) and 3D ultrasound of the prostate emerging as alternative tools for IGRT it is important to assess the safety of using transrectally implanted fiducial markers.

Therefore the aim of our study was to evaluate the rate of infection from transrectal fiducial marker implantation for prostate IGRT whilst also minimizing the biases which can affect studies of this nature.

## Methods

All patients at our centre who underwent gold seed fiducial marker implantation for prostate IGRT between January 2012 to December 2013 were identified through a local prospectively maintained database. The patients had the procedure done by their local urologists. All patients were prescribed prophylactic fluoroquinolone antibiotics peri-procedure, and rectal enemas were not always used. Under local anaesthetic, three gold markers of 1.0x3.0 mm each were implanted into the prostate base, apex and contralateral midzone with TRUS guidance. Our primary endpoint was the rate of urosepsis requiring hospital admission, with a secondary endpoint as the rate of urinary tract infection. This study received approval from a local human research ethics committee, the Hunter New England Human Research Ethics Committee (HREC Reference no: 14/02/19/5/09).

### Questionnaire outline

Questionnaires were sent by post in mid-March 2014. This was followed-up with a phone call reminder if a reply had not been received at 3 weeks. The questionnaire was 1-page long consisting of 5 questions (Additional file 1) directed at infective symptoms and any related treatment received including antibiotics and/or hospital admissions following the procedure. Each question required a “Yes” or “No” answer, mirroring the approach of one of the largest previous reports [1]. There was also space for free text comments, and many of the respondents took up this option. All responses were collected and entered into a database. For those that answered yes to receiving antibiotic and/or hospital admissions, we corroborated the information with their medical records and also with their general practitioners where required. A consensus panel then reviewed the medical information of these patients, and where it was clear that the hospital admission was not related to the fiducial marker procedure, the patient was excluded from the analysis. Descriptive statistics were prepared to summarize the data using Excel (Microsoft, Seattle).

## Results

A total of 359 patients were identified from our database and questionnaires were sent out to all of these patients’ last recorded address. We received 297 responses (82.7%), of which 12 declined participation. The final reply was received approximately 6 weeks after mail out. Therefore 285 (79.4%) completed questionnaires were returned.

Seventy-seven (27.0%) men reported experiencing symptoms of increased urinary frequency or dysuria and 33 (11.6%) men experienced episodes of chills and fever after the procedure.

Twenty-two (7.7%) men answered yes when asked if they were treated for a urinary infection with antibiotics after the procedure. For 16 of the 22 patients we were able verify this through their medical records and therefore at the minimum, the rate of infectious complication requiring antibiotic treatment in our study is 5.6%.

Eight (2.8%) patients reported a hospital admission for infective complications related to the fiducial implantation procedure. We were able to obtain medical records of the hospital admission for 5 of the 8 patients. Therefore the minimum rate of hospital admission for infective complications in our study is 1.8%. All 5 patients presented within 5 days of the procedure and one patient required 2 hospital admissions for persistent infection. One of the 8 patients had urosepsis after his initial TRUS biopsy, but we were not able to verify if he had a hospital admission following his fiducial marker procedure. Twelve (4.2%) patients reported recurrent urinary infections requiring antibiotics. Three of the twelve patients developed chronic prostatitis at 7 to 12 months after completion of their radiotherapy requiring prolonged courses of antibiotics.

*E.Coli* was the most common organism grown in those with a positive urine culture, although we were not able to obtain information on the sensitivities to fluoroquinolones for all of them. Other organisms isolated include *enterococcus* and *proteus mirabilis*.

## Discussion

Although fiducial markers have been widely accepted as standard for prostate IGRT, there is a lack of quality evidence relating to their safety. Studies evaluating the safety of the implantation of the fiducial markers are all single arm case series, with significant heterogeneity across the studies (Table 1) [1-4]. Several factors may explain the higher rate of hospital admissions reported in our study (1.8-2.8%) compared to the published literature of between 0 to 1.3%. Similarly, the rate of urinary infections requiring antibiotic therapy in our series at 5.6 to 7.7% is higher compared to reported rates in the literature (1.9-2.2%). These studies all evaluated the toxicities and complications through the use of a questionnaire. This relies heavily on the patients’ accurate recollection of symptoms and events. The accuracy decreases as the recall period increases. This can lead to over or under-reporting. In our study, we chose to include patients who underwent this procedure within the last 2 years to minimise the possibility of recall bias. Most of the published studies also asked about a broad range of symptoms and toxicities. Different scales and scoring systems were employed in the studies. In one study, the questionnaire was up to 10 pages long. This increases the non-response rate and can further compound the recall bias. We chose to limit our questionnaire to a single page, directed only to the infective complications. We were also able to validate the patient reported infectious complications and the treatment received from their medical records for the majority of patients in our study. For these reasons, it is possible previous series have underestimated the true incidence of infective complications associated with TRUS guided gold fiducial insertion. Nonetheless, our study being retrospective is likely to still suffer from similar limitations of other studies of this nature (Table 1), with the possibility of under or overestimating the complications.

**Table 1:** **Studies assessing complication rates from fiducial marker implantation for prostate image guided radiotherapy**

**Study**	**Patient numbers**	**Infective complications**
**Gill et al.** [1]	234	3 patients (1.3%) required admission for sepsis
		1 patient (0.4%) had Grade 4 septicaemia.
**Langenhuijsen et al.** [2]	209	4 patients (1.9%) with fever received additional antibiotics.
		There were no hospital admissions.
**Igdem et al.** [3]	135	3 patients (2.2%) developed symptomatic urinary infection with fever and received antibiotics.
		There were no hospital admissions.
		1 (0.7%) patient developed asymptomatic bacteriuria resistant to antibiotic therapy.
**Moman et al.** [4]	402 had transrectal FM implantation	2 (0.5%) developed urosepsis (Grade 3 toxicity)
	512 had transperineal FM implantation	No grade 3 or 4 toxicity.
**Loh et al.**	285	22 patients (7.7%) received antibiotic treatment for a urinary infection
		8 patients (2.8%) required hospital admission for urosepsis.
**TRUS biopsy series**		
**Wagenlehner et al.** [10]	521	5.2% had symptomatic urinary tract infection
		3.1% required hospital admissions for infectious complications

FM = fiducial marker.

TRUS = Transrectal ultrasound guided.

There is more significant data on infectious complications available in the transrectal prostate biopsy setting. The rate of symptomatic and febrile urinary infections following transrectal prostate biopsy in large multi-institutional studies is approximately 4%, leading to hospital admission in up to 3.1% of patients [7]. Although local factors in gold seed implantation may have had an impact on the higher infection rates we report compared to other similar series, we note that our results are comparable to this transrectal biopsy data. Potential risk factors for post-biopsy infections have been identified in a number of studies [6,8-10]. They include a high comorbidity index, exposure to antibiotics within 6 months of biopsy, hospital employment and recent international travel. The presence of fluoroquinolone resistant organisms in the faecal reservoir is a significant risk factor for post biopsy infection [5,11,12]. Fluoroquinolone resistant bacteria have been detected in about 50-90% of cases with post-TRUS biopsy infections, with emerging reports of multidrug resistance. Currently the American Urological Association best practice guidelines still recommend fluoroquinolones or 1^st^-3^rd^ generation cephalosporins as antimicrobials of choice for prophylaxis. The guidelines call for the use of an alternative anti-microbial in patients with risk factors for infectious complications following prostate biopsy. However, the regular use of broad-spectrum antibiotics is not the ideal solution, as this will possibly lead to even more resistance. The use of rectal enemas before biopsy is under investigation, although studies have so far not shown significantly reduced infection rates [13,14]. Pre-procedural rectal swabs to guide prophylactic antibiotic therapy have shown promising early results [15]. Transperineal prostate biopsy is also being explored as a method to avoid inoculation of rectal flora into the prostate. Data suggests a negligible rate of sepsis with this approach, although a general anaesthetic is often required [16]. It would seem prudent to incorporate some of these observations into the evolving understanding of fiducial marker placement.

There is a paucity of evidence and guidelines to identify individuals at risk and the prevention of infectious complications following fiducial marker implantation. Given the similarity of the two procedures, current strategies have been adopted from the transrectal prostate biopsy setting. Evidence shows that in men with prostate cancer on active surveillance, serial repeat prostate biopsies are associated with a significant risk of infectious complications [17]. For every previous biopsy the odds of an infection increased 1.3 times [17]. There is no data currently to show the same with fiducial marker implantation following an initial prostate biopsy, but given the similarity of the procedures, the prostate biopsy data may also hold true in this setting. It is also not known if the acquired infections post fiducial marker implantation are more difficult to treat and harder to eradicate, as unlike the post biopsy setting, the fiducial markers could potentially serve as an enduring nidus of infection within the prostate. One patient in our study who grew a fluoroquinolone resistant *E.Coli* required 2 hospital admissions and long-term antibiotics for persistent infection. He eventually required a trans-urethral resection of necrotic prostate tissue, which is likely to have been a consequence of his chronic infections rather than solely due to his radiotherapy. Transperineal fiducial marker implantation may lead to less infective complications although there is still a risk of bleeding and pain.

Chronic prostatitis following fiducial marker implantation is not easily recognisable in a clinical setting. It is a diagnosis of exclusion, with sterile pyuria often seen in these patients. The prolonged symptoms can impact on the patient’s quality of life. There is scarce literature on chronic prostatitis in this setting, likely reflecting the under-recognition of the condition in clinical practice and the difficulties in establishing a definitive diagnosis.

There are costs to both the health provider and to the patients associated with this procedure. In Australia, the minimum cost for fiducial marker implantation is just over three hundred dollars per patient. This is excluding the cost of the fiducial markers themselves which is approximately one hundred and seventy dollars. The cost also increases if the patient requires general anaesthetic. For the patient, the procedure requires an extra appointment and if pre procedural infection screening becomes routine, this will add to the number of visits. In some cases co-ordination of the temporary cessation of their anti-coagulation therapy is also required. One patient in our study suffered a stroke as a consequence of subtherapeutic warfarin levels following recommencement after his procedure. Although this event is uncommon, the consequences for the patient when it occurs can be disastrous. For patients who had developed a complication following their prostate biopsy, this exposes them again to another potential risk of complication.

The superiority of fiducial markers versus bony landmark based IGRT in prostate motion management is widely accepted [18]. The use of daily fiducial marker based IGRT is also associated with lower rates of patient reported rectal toxicity compared to a cohort managed with bony matching [19]. Non-invasive methods to localize the prostate gland such as 3D ultrasound [20] and CBCT [21] with soft tissue matching have been assessed as alternate tools for image guidance. A study comparing soft tissue matching using CBCT versus orthogonal megavoltage image matching with fiducial markers showed that in less than 5% of fractions a disagreement of >5mm was observed. The authors hypothesized that the most extreme differences in the shifts were likely due to higher interobserver variability in locating the prostate on CBCT images [21]. A second series also demonstrated higher interobserver variability with CBCT soft tissue matching compared with fiducial markers, and recommended that this should be taken into consideration in the margin generation [22]. Nonetheless, intraprostatic fiducials do allow greater accuracy of IGRT compared with soft-tissue based guidance with CBCT or ultrasound. As a consequence of this, one possibility may be to use fiducial markers mainly where higher accuracy is important such as stereotactic prostate radiotherapy, and CBCT in situations such as history of urosepsis following biopsy or anticoagulation. Such risk adaption of the method of IGRT would potentially reflect the wider movement towards tailored treatments in oncology.

## Conclusion

Fiducial marker implantation for prostate IGRT is associated with a small percentage of patients experiencing moderate to severe complications requiring further medical interventions. However, the methods used to assess for toxicities are insufficient to adequately capture complications such as chronic prostatitis. Alternative non-invasive methods for image guidance such as CBCT and ultrasound might be considered for conventionally fractionated prostate radiotherapy.

## Additional file

Additional file 1:
**Example of the questionnaire.**
