# Supplementary material for: Infections after fiducial marker implantation for prostate radiotherapy: are we underestimating the risks?
Source: Radiat Oncol. 2015 Feb 13;10:38. doi: 10.1186/s13014-015-0347-2 (PMC4333155; doi:10.1186/s13014-015-0347-2)
Supplement: Additional file 1: — Example of the questionnaire. [file 13014_2015_347_MOESM1_ESM.docx]

Additional file 1. Example of the questionnaire

| **Rate of infection with gold fiducial marker implantation**  **for prostate image-guided radiotherapy survey**  **AFTER** the insertion of the gold fiducial markers (or gold seeds) for your prostate radiotherapy, did you experience any of the following within **one month** after the procedure:   1. Did you experience symptoms of increased frequency of urination or a burning sensation when you passed urine?   **YES** **NO**   1. Did you experience any episodes of chills and fevers?   **YES** **NO**   1. Were you treated for a urinary infection with antibiotics within a month **after** the procedure? (This does not include the antibiotics that were given for the few days around the gold seed procedure).   **YES** **NO**     1. Did you require any hospital admission for complications related to the gold seed procedure, including a urinary infection?   **YES** **NO**   1. Have you had recurrent urinary infections requiring antibiotics since you had the gold seeds inserted?   **YES** **NO** |
| --- |
